# Characterization and partial purification of *Candida albicans *Secretory IL-12 Inhibitory Factor

**DOI:** 10.1186/1471-2180-8-31

**Published:** 2008-02-19

**Authors:** Mingyue Wang, Pranab K Mukherjee, Jyotsna Chandra, Ali Abdul Lattif, Thomas S McCormick, Mahmoud A Ghannoum

**Affiliations:** 1 Center for Medical Mycology, Department of Dermatology, University Hospitals of Cleveland and Case Western Reserve University, Cleveland, Ohio, USA; 2 Department of Dermatology, University Hospitals of Cleveland and Case Western Reserve University, Cleveland, Ohio, USA; 3 Research Center for Medical Mycology, Department of Dermatology, Peking University First Hospital, Beijing, China

## Abstract

**Background:**

We have previously shown that supernatant from *Candida albicans *(CA) culture contains a Secretory Interleukin (IL)-12 Inhibitory Factor (CA-SIIF), which inhibits IL-12 production by human monocytes. However, the effect of CA-SIIF on secretion of other cytokines by monocytes is unknown, and detailed characterization of this factor has not been performed.

**Results:**

In this study, we demonstrate that the IL-12 inhibitory activity of CA-SIIF was serum-independent, based on the reduction of IL-12 levels in monocytes stimulated under serum-independent conditions. The minimal inhibitory dose of CA-SIIF was found to be 200 μg/ml. Investigation of CA-SIIF's effect on macrophages IL-12 production *in vitro *and *in vivo *also showed that CA-SIIF inhibited IL-12 production by murine macrophages both *in vitro *(from 571 ± 24 pg/ml to 387 ± 87 pg/ml; P = 0.05) and *in vivo *(from 262 ± 6 pg/ml to 144 ± 30 pg/ml; *P *< 0.05). In addition to IL-12, cytokine array analysis revealed that CA-SIIF induced differential production of other cytokines also. In this regard, reduction in levels were observed for IL-8, IL-10, IL-13, monocyte chemoattractant protein (MCP)-1, MCP-2, macrophage inflammatory protein (MIP)-1, RANTES, etc. In contrast, levels of other chemokines e.g. MCP-4, MIF and MIP-3α (*P *< 0.05) were increased. We also found that CA-SIIF suppressed the maturation of human monocytes to dendritic cells (CD1a expression = 13 ± 3% vs 36 ± 2% of the control; *P *< 0.01). Next, to identify the biochemical nature of CA-SIIF, we separated this factor into a Concanavalin A (ConA)-binding glycoprotein fraction (CA-SIIF-GP) and a non-ConA-binding protein fraction (CA-SIIF-NGP) using ConA affinity chromatography. Both fractions were then tested for this inhibitory effect on human monocyte IL-12 production. CA-SIIF-GP produced a higher inhibitory effect on IL-12 production compared to CA-SIIF-NGP and CA-SIIF crude (*P *< 0.01), proving that CA-SIIF is a glycoprotein in nature.

**Conclusion:**

CA-SIIF is a glycoprotein which exhibits serum-independent inhibition of IL-12 production from monocytes *in vitro *and *in vivo*, and also modulates differentiation of monocytes into dendritic cells. These results suggest important role for CA-SIIF in interactions of *C. albicans *with the host immune system.

## Background

Infections due to the human pathogenic fungus *Candida albicans *are a major cause of morbidity and mortality in immunocompromised patients [1] and one of the most common causes of nosocomial bloodstream infections [2]. Host response to *Candida *infection is a complex interplay between innate and adaptive immunity, and usually provides a sufficient defense against microbes in healthy individuals but not in immunocompromised ones. The first line of defense against *Candida *infections are immune cells involved in the innate immune response, including monocytes, which can differentiate into macrophages [3] or dendritic cells [4] under various conditions.

The defense mechanisms activated by these immune cells involve stimulation of pro-inflammatory cytokines like interleukin-12 (IL-12) and/or inhibition of anti-inflammatory cytokines (e.g., IL-10) by the host monocytes/macrophages. Among those cytokines, IL-12 plays an important role in differentiating T cells and activating Natural Killer (NK) cells, both of which produce high levels of IFN-γ, leading to protective cell-mediated immunity against *Candida *infection [5].

A common mechanism by which microbial pathogens overcome host immune response is by suppressing production of pro-inflammatory cytokines like IL-12 [6,7]. Previously, we showed that a Secretory IL-12 Inhibitory Factor produced by *C. albicans *(CA-SIIF) inhibits IL-12 production by human monocytes [8]. We hypothesized that CA-SIIF induced differential production of other cytokines, and that CA-SIIF is a glycoprotein. To test this hypothesis, in this study, we determined the effect of CA-SIIF on: (a) profile of cytokines/chemokines produced by monocytes exposed to this factor, (b) differentiation of monocytes to dendritic cells, and (c) IL-12 production by murine macrophages *in vitro *and *in vivo*. Furthermore, we also performed concanavalin A (ConA) affinity chromatography to isolate the glycoprotein fraction of CA-SIIF and determined whether the IL-12 inhibitory activity is mediated by this fraction. We found that, in addition to its effect on IL-12 production, in the presence of CA-SIIF, stimulated monocytes produce different levels of GRO (Growth Related Oncogene), IL-8, IL-10, IL-13, MCP (Monocyte Chemoattractant Protein)-1, MCP-2, MIP (Macrophage Inflammatory Protein)-1Δ, RANTES (Regulated upon activation, normal T-cell expressed, and presumably secreted), Leptin, Eotaxin-2, LIF (Leukemia Inhibitory Factor), TIMP (Tissue inhibitor of metalloproteinases)-2, MCP-4, MIF (Macrophage Migration Inhibitory Factor) and MIP-3α. Additionally, CA-SIIF also inhibited differentiation of monocytes into dendritic cells. CA-SIIF also inhibited production of IL-12 by murine macrophages, both *in vitro *and *in vivo*. The IL-12 inhibitory activity of CA-SIIF was localized to the ConA-based affinity purified glycoprotein fraction, suggesting that a glycoprotein moiety mediates the inhibitory activity of CA-SIIF. The present studies show that CA-SIIF is a glycoprotein, and demonstrated that it can induce differential production of several cytokines in addition to IL-12, and that its mechanism of action may be mediated by inhibition of monocyte differentiation.

## Methods

### Fungal organisms and culture conditions

*C. albicans *(strain SC5314) was used in this study to obtain CA-SIIF. A non-pathogenic yeast, *Saccharomyces cerevisiae *(strain MRL138), was used as a comparator. Cells were grown in Yeast Nitrogen Base (YNB) (BD Biosciences, Sparks, MD) for 18 h, and used as described below for collection of CA-SIIF. Frozen stocks were maintained for these cultures at -80°C.

### Collection of CA-SIIF

Yeast strains grown in YNB were subcultured (3 × 10^5 ^cells/ml) in RPMI 1640 medium (Mediatech, Herndon, VA) in a shaking incubator at 37°C in 4 L volume flasks for 20 hours. The supernatants from the above culture (or RPMI medium as controls) were collected and transferred to Centricon filters with a molecular weight cut-off (MWCO) of 30 kDa (Millipore, Bedford, MA). Protein concentrations were measured using a BCA Protein Assay Kit (Pierce, Rockford, IL). CA-SIIF was filtered through a 0.22 μm filter unit (Millipore, Bedford, MA), aliquoted, concentrated and stored at -80°C until use [8].

### Isolation of human monocytes

Monocytes were obtained from fresh human peripheral blood of healthy adult volunteers as described previously [9]. Volunteers participated in this study with informed consent after approval of the protocol by the Institutional Review Board (IRB) of Case Western Reserve University (CWRU) and University Hospitals of Cleveland (UHC). Briefly, 240 ml heparinized blood was centrifuged over a Ficoll gradient-1077 (Sigma-Aldrich, St. Louis, MO), and adherent cells from the buffy coat were harvested and isolated by negative selection using an antibody cocktail (StemCell Technologies, Vancouver, Canada). The cell mixture was then passed through a Magnetic Cell Separation (MACS) column against a MACS magnet (Miltenyi Biotec, Auburnm, CA) to obtain the purified monocytes. The purity of the monocytes was determined by flow cytometry using CD14 positive staining and ranged between 85%–95%.

### Co-culture of monocytes and yeast supernatants

Immediately after purification, 1 × 10^6 ^monocytes in 1 ml complete media (RPMI-1640 with 10% FBS and 1% Penicillin/Streptomycin) were incubated with 50 μl CA supernatants (50 μg, 100 μg, 200 μg and/or 300 μg, depending on each experiment) and primed with 5 ng/ml IFN-γ at 37°C with 5% CO_2 _for 16 hours, followed by another 24 hours of 50 ng/ml LPS stimulation [10]. Supernatants of cell culture were collected and cytokine levels were measured as described below.

### *In vitro *assay to evaluate inhibition of IL-12 production by murine macrophages

For *in vitro *studies, peritoneal macrophages were extracted from female C57BL/6 mice 6–8 weeks of age elicited by 1 ml of 3% thioglycollate (TG), and then 2 × 10^6 ^cells in 1 ml complete media were incubated with 500 pg/ml IFN-γ and 50 ng/ml LPS, in the presence 1 mg CA-SIIF or media control. Finally, IL-12 levels were measured by ELISA as described below. Experiments complied with Institutional Animal Care and Use Committee (IACUC) guidelines of CWRU.

### *In vivo *assay to evaluate inhibition of IL-12 productions in murine macrophages

**Figure 1:**
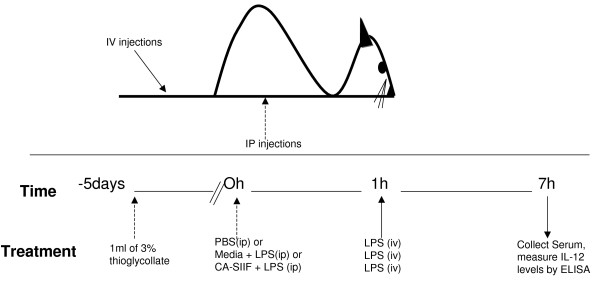
Schematic showing steps involved in the *in vivo *studies with CA-SIIF.

IL-12 production *in vivo *was optimized using female C57BL/6 mice of 6–8 weeks age as above pretreated for 5 days with TG peritoneally (ip) and combining a priming ip injection with 100 ng LPS with an iv injection of 1 μg LPS 1 h later. 1 mg CA-SIIF or media control was injected prior to LPS, and six hours later, serum was collected (please see schematic, Figure 1). Serum IL-12 levels were then measured by ELISA as described below. Experiments complied with IACUC guidelines of CWRU.

### Cytotoxicity of CA-SIIF and human cell viability

To exclude the possibility that CA-SIIF was potentially toxic to mice, up to 1 mg of CA-SIIF was injected intravenously into both BALB/c and C57BL/6 mice (5 per group) and those mice were monitored daily over a 2-week period. *In vitro *induction of Red Blood Cell (RBC) lysis at different CA-SIIF protein concentrations (100–1200 μg) was also determined, with amphotericin B (AmB, with known toxicity against RBCs) serving as a positive control, while fluconazole (FLU, an antifungal with very safe toxicity profile) as negative control. Human cell viability was also confirmed by trypan blue staining indicating over 75% viability in each experiment.

### Quantitation of IL-12p70

The levels of IL-12p70 were determined by Enzyme-Linked Immunosorbent Assay (ELISA) using a Human IL-12 (p70) kit (BD Biosciences, San Diego, CA). Quantities of IL-12 p70 were expressed as pg/ml. The sensitivity of the ELISA was ≥ 4 pg/ml. All assays were performed in duplicates or triplicates.

### Determination of cytokine/chemokine profile

Cytokines and chemokines were examined via multiplex spot blot array for "inflammatory cytokines" to assess the effect of CA-SIIF on monocytes. To determine changes in the overall cytokine/chemokine profile following CA-SIIF exposure to monocytes, we measured the levels of cytokine response following monocyte stimulation by IFN-γ and LPS in the presence or absence of CA-SIIF using the Human Cytokine Antibody Array 5 (Ray Biotech, Inc., Norcross, GA.) [11]. Briefly, each membrane was placed in an eight-well tray and was blocked with blocking buffer according to manufacturer's instructions. The membrane was incubated with 1 ml of supernatant obtained from stimulated monocytes treated with or without CA-SIIF, followed by biotin-conjugated anti-cytokine antibody treatment. Next, membranes were washed and incubated with horseradish peroxidase (HRP)-conjugated streptavidin and the signals were captured using a chemiluminescent phosphoimager (VersaDoc, BioRad Laboratories, CA). Cytokines spots were quantified using a densitometer (BioRad VersaDoc, CA) and relative values were calculated as percentage with respect to internal positive controls (used as 100%) on each cytokine array membrane. Cytokines that differed significantly from the internal controls were considered to be cytokines of interest.

### Differentiation from monocytes into dendritic cells

Monocytes (5 × 10^5^/ml) in complete media (described above) were incubated with GM-CSF (1000 u/ml) and IL-4 (500 u/ml) for 5 days with or without addition of 30 μl (500 μg) CA-SIIF. After 3 washes, the monocyte-derived dendritic cells were first incubated with purified heat-aggregated human IgG (2.5 g/ml, Sigma-Aldrich, St. Louis, MO) for 20 min on ice. Cells were then directly stained with FITC-conjugated anti-CD14 (Dakocytomation, Inc. Carpinteria, CA), PE-conjugated anti-CD1a (Caltag Laboratories, Burlingame, CA) and their appropriate isotype controls. The stained cells were analyzed by flow cytometry by using WinList software (Verity Software House, Topsham, ME). Positive staining cells were expressed as a percentage after subtraction of cells in the same gate with isotype controls.

### Glycoprotein isolation by ConA affinity chromatography

To separate glycoprotein and non-glycoprotein fractions of CA-SIIF, lectin affinity chromatography was performed using a concanavalin A (ConA)-based Glycoprotein Isolation Kit (Pierce, Rockford, IL). Briefly, unfractionated CA-SIIF containing up to 1.5 mg of total protein was first diluted with the Binding/Wash Buffer and applied to the ConA resin bed. Following incubation for 10 minutes, the resin was washed and the bound glycoproteins were eluted. After dialysis using 10 kDa MWCO Slide-A-Lyzer Dialysis Cassettes (Pierce, Rockford, IL), samples were further processed by 10 kDa MWCO Centricon filter (Millipore, Bedford, MA). Finally, protein concentration levels of each fraction were measured and sample volumes were concentrated as described above.

### Statistical analysis

Results were expressed as mean ± standard error (SEM) for N number of replicated experiments. Statistical significance of difference between groups was determined by two-tailed Student's *t *test. *P *value of < 0.05 was considered significant.

## Results

### IL-12 inhibitory activity of CA-SIIF is serum-independent, and induced in a dose-dependent manner

**Figure 2:**
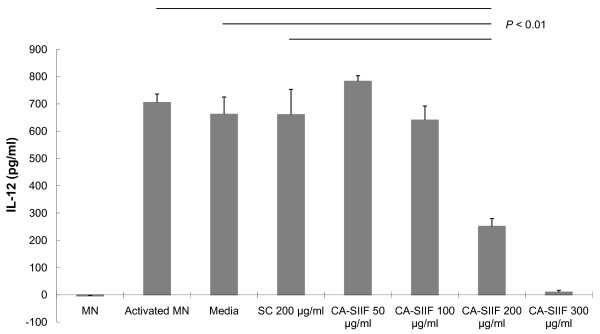
**CA-SIIF inhibited induced IL-12 production by human monocytes in a dose dependent manner**. 1 × 10^6^/ml human monocytes were primed with IFN-γ for 16 hours and activated by LPS for 24 h with supernatants (>30 kDa) from *C. albicans *or from *S. cerevisiae *or media control collected under same conditions. The supernatants were then measured for IL-12 p70 production. Monocytes (MN), monocytes activated by IFN-γ and LPS, 30 kDa MWCO media and supernatant from *S. cerevisiae *served as various controls. MN: monocytes without activation. Activated MN: monocytes activated by IFN-γ and LPS. CA-SIIF: activated MN cultured with supernatants from *C. albicans*. SC: activated MN cultured with supernatants from *S. cerevisiae*. *n *≥ 3.

Previously, we showed that CA-SIIF is secreted by *C. albicans *grown in the presence of fetal bovine serum (FBS), and that this factor can inhibit IL-12 production by monocytes activated by heat-killed *C. albicans *(HKCA) cells [8]. Since it is possible that factors present in FBS and/or heat-killed *C. albicans *cells may influence the inhibitory activity of CA-SIIF, we determined whether CA-SIIF obtained from *C. albicans *grown in serum-free medium also inhibited IL-12 production by monocytes. CA-SIIF was collected from *C. albicans *cultures grown for 20 h in serum-free media and added in different concentrations (50, 100, 200 and 300 μg/ml) to monocytes co-cultured with IFN-γ and LPS. We found that CA-SIIF obtained from *C. albicans *grown in serum-free medium also inhibited IL-12 production by monocytes. Furthermore, this inhibition was dose-dependent, with the highest inhibition observed for 300 μg/ml (IL-12 level = 10 ± 7 pg/ml) and the lowest for 200 μg/ml *C. albicans *supernatant (IL-12 level = 251 ± 28 pg/ml), compared to untreated monocytes (705 ± 31 pg/ml,*P *< 0.01 for all comparisons). At lower concentration (100 μg/ml), this supernatant exhibited a trend to decrease IL-12 levels, but the decrease was not statistically significant. As expected, supernatants obtained from *S. cerevisiae *(SC) (661 ± 93 pg/ml) or RPMI-1640 media controls (662 ± 63 pg/ml) did not induce significant inhibition of IL-12 levels (*P *< 0.01, Figure 2). These results demonstrated that CA-SIIF is produced by *C. albicans *cells in a serum-independent, but dose-dependent manner.

### CA-SIIF inhibits IL-12 production from murine macrophages *in vitro *and *in vivo*

**Figure 3:**
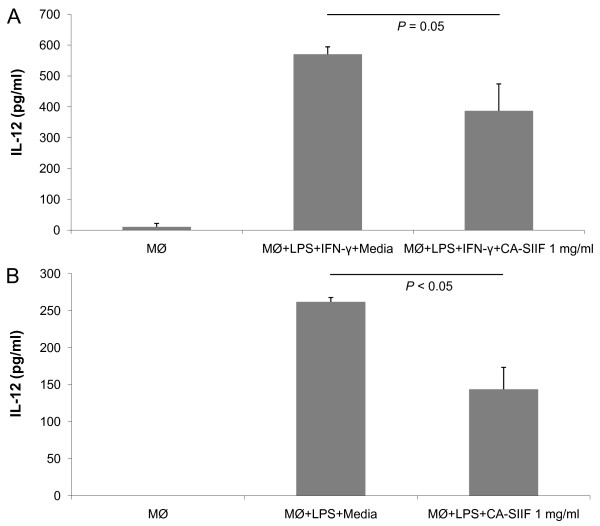
**CA-SIIF inhibits macrophage IL-12 production both (A) *in vitro *and (B) *in vivo *in a murine model**. **(A) **murine peritoneal Macrophages (MØ) were elicited by TG treatment and collected from the peritoneum of C57Bl/6 mice 5 days after elicitation. 2 × 10^6^/ml macrophages were cultured in the presence or absence of LPS (50 ng) and IFN-γ (0.5 ng) stimulation for 16 h with or without 1 mg CA-SIIF. Next, IL-12 levels were measured in the supernatant by ELISA using antibodies specific for murine IL-12 p70. **(B) **LPS (100 ng) was administered intraperitoneally immediately followed with or without 1 mg CA-SIIF in mice 1 h prior to intravenous injection of LPS (1 μg) to stimulate IL-12 production. Serum IL-12 levels were then measured. Macrophages alone served as controls for base line IL-12 production.

Monocytes differentiate into macrophages and are the first line of defense against *Candida *infection [12]. Therefore, we determined whether CA-SIIF affects IL-12 production by murine macrophages. Macrophages were isolated from the peritoneum of C57BL/6 mice following TG elicitation, and then exposed to CA-SIIF in combination with IFN-γ/LPS and the levels of IL-12 were determined. We found that macrophages co-incubated with CA-SIIF produced 32% less IL-12 than those grown in its absence (IL-12 level = 571 ± 24 pg/ml vs. 387 ± 87 pg/ml, respectively; *P *= 0.05, Figure 3A). Next, we established a murine model to investigate the influence of CA-SIIF on IL-12 production *in vivo*. In this regard, CA-SIIF toxicity assays in mice showed no signs of toxicity after injection of up to 1 mg CA-SIIF, over a 2 week period. RBC lysis induction tests at various CA-SIIF concentrations (100–1200 μg) were also negative (data not shown). IL-12 production *in vivo *in the presence or absence of CA-SIIF was examined using C57BL/6 mice pretreated for 5 days with TG peritoneally in combination with a priming ip injection of LPS (100 ng) and another iv LPS (1 μg) injection 1 h later. Prior to the first LPS treatment, CA-SIIF was injected ip, and six hours later, serum was collected (Figure 1). Our data showed that the level of IL-12 detected in the sera of mice treated with CA-SIIF was significantly decreased from 262 ± 6 pg/ml to 144 ± 30 pg/ml (*P *< 0.05) (Figure 3B). These results demonstrated that CA-SIIF can decrease IL-12 production by murine macrophages both *in vitro *and *in vivo*.

### CA-SIIF alters the balance of pro- and anti-inflammatory cytokines

**Figure 4:**
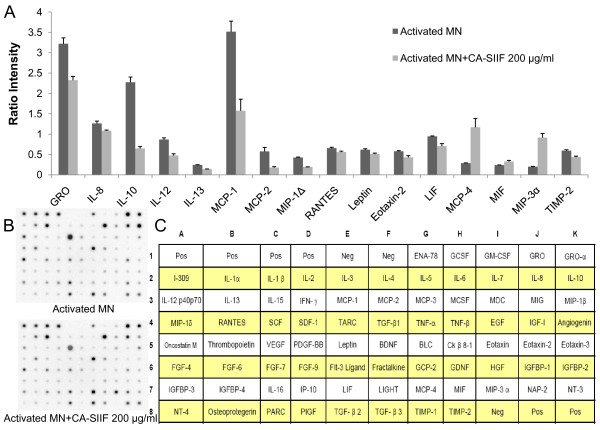
**Cytokine profiles of supernatants obtained from IFN-γ and LPS stimulated monocytes cultured in presence or absence of CA-SIIF**. 1 × 10^6^/ml human monocytes were grown in the absence or presence of CA-SIIF with IFN-γ and LPS for 40 h, their supernatants were collected, and the cytokines present in these supernatants were measured using the preprinted human cytokine antibody arrays 5 (Ray Biotech, Inc.). **(A) **differentially expressed cytokines/chemokines with *P *value less than 0.05 (*n *= 3). **(B) **representative images of cytokine array membranes. **(C) **cytokine map of the membrane used, showing location of cytokines on the membrane.

Although CA-SIIF was shown to inhibit monocyte IL-12 production, its effect on production of other cytokines/chemokines was unknown. Since microbial pathogens are known to overcome host immune responses by modulating levels of pro- and anti-inflammatory cytokines, we used membrane arrays to evaluate cytokine/chemokine profile of monocytes exposed to CA-SIIF. We found that in addition to decreased IL-12, CA-SIIF also inhibited production of other cytokines/chemokines including GRO, IL-8, IL-10, IL-13, MCP-1, MCP-2, MIP-1Δ, RANTES, Leptin, Eotaxin-2, LIF and TIMP-2 by monocytes. In contrast, other chemokines like MCP-4, MIF and MIP-3α were increased significantly (*P *< 0.05, Figure 4). These studies suggested that inhibition of pro-inflammatory cytokines/chemokines and increase of anti-inflammatory ones (e.g. MIF) may be one of the mechanisms by which the inhibitory activity of CA-SIIF is mediated. Alternatively, the balance of pro- and anti- inflammatory cytokines may be changing dynamically following CA-SIIF treatment. For example, IL-10, an anti-inflammatory cytokine was decreased following CA-SIIF treatment, perhaps following an increase in levels of IL-10 prior to our sampling point.

### CA-SIIF inhibits differentiation of monocytes into dendritic cells

**Figure 5:**
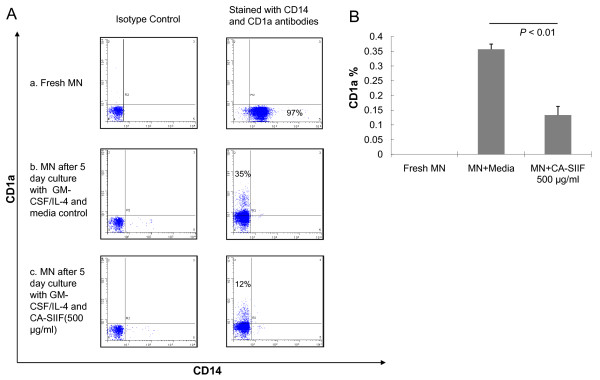
**CA-SIIF dramatically reduces CD1a expression on human monocyte-derived dendritic cells**. **(A) **Impact of CA-SIIF on GM-CSF/IL-4 induced monocyte-derived dendritic cells. (a) CD14 expression on fresh monocytes, (b) CD1a expression on monocytes after 5 day culture with GM-CSF/IL-4 in absence of CA-SIIF, (c) CD1a expression on monocytes after 5 day culture with GM-CSF/IL-4 in presence of CA-SIIF. Flow histograms are representative of 3 independent experiments. **(B) **Cumulative dramatic reduction of CD1a expression on human monocytes-derived dendritic cells cultured with CA-SIIF. Cell density: 5 × 10^5^/ml. *n *= 3.

Our previous data suggested CA-SIIF IL-12 inhibition involves the Extracellular signal-regulated Kinase (ERK) Mitogen-Activated Protein Kinase (MAPK) signaling pathway [8]. Since ERK and p38 MAPK signaling pathways are known to differentially regulate the maturation of monocyte-derived human dendritic cells [13], we hypothesized that CA-SIIF inhibits differentiation of human monocytes into dendritic cells. As shown in Figure 5, flow cytometry analysis revealed significantly reduced expression of CD1a (a surface marker specific for dendritic cells) in monocytes incubated with CA-SIIF compared to control media (13 ± 3% vs 36 ± 2%; *P *< 0.01). These studies demonstrated that CA-SIIF inhibits differentiation of human monocytes to dendritic cells.

### Glycoproteins present in CA-SIIF mediate its IL-12 inhibitory activity

**Figure 6:**
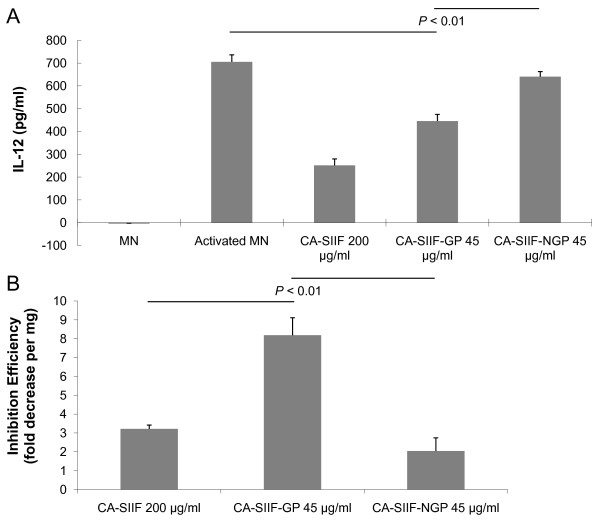
**Glycoprotein fraction of CA-SIIF demonstrated higher inhibitory effect on human monocytes IL-12 production**. CA-SIIF was passed through a ConA lectin affinity column. Both resulting fractions and CA-SIIF crude were tested for the effect on IL-12 production of 1 × 10^6^/ml human monocytes. **(A) **glycoprotein fraction (CA-SIIF-GP) successfully achieved higher inhibitory effect than the non-glycoprotein (CA-SIIF-NGP) fraction (*P *< 0.01). **(B) **Inhibition efficiency of CA-SIIF-GP fraction is significantly higher compared to CA-SIIF crude and CA-SIIF-NGP (*P *< 0.01). Fold decrease was calculated by decrease of the IL-12 level by percentage; inhibition efficiency was determined by evaluating the fold decrease of IL-12 level/protein dose ratio.

In the previous study, we showed that CA-SIIF contains a carbohydrate component [8]. In this study, we determined whether CA-SIIF is a glycoprotein using affinity chromatography based on binding to ConA (a lectin that binds specifically mannosyl/glucosyl residues) [14]. We separated ConA-binding glycoprotein fraction (CA-SIIF-GP) and non-ConA-binding non-glycoprotein fraction (CA-SIIF-NGP) from CA-SIIF, and determined the effect of these fractions on IL-12 production by activated monocytes. Our results showed that activated monocytes grown in presence of CA-SIIF-GP produced significantly less IL-12 than those grown in its absence (IL-12 level = 446 ± 29 pg/ml vs. 705 ± 31 pg/ml, respectively, P < 0.01). In contrast, addition of CA-SIIF-NGP was unable to induce a similar reduction in monocytic IL-12 levels (IL-12 level = 641 ± 22 pg/ml vs. 705 ± 31 pg/ml; *P *< 0.01, Figure 6A). To further demonstrate the enhanced effect of CA-SIIF after purification, inhibition efficiencies were calculated based on the fold decrease of monocyte IL-12 level (per mg protein). As can be seen in Figure 6B, CA-SIIF-GP exhibited a significantly higher IL-12 inhibition efficiency (fold decrease = 8.2 ± 0.9) compared to crude CA-SIIF supernatant or CA-SIIF-NGP (fold decrease = 3.2 ± 0.2 or 2.0 ± 0.7 respectively; *P *< 0.01). These studies demonstrated that partial purification of CA-SIIF based on glycoprotein properties increased CA-SIIF activity and suggested that this inhibitory activity of CA-SIIF is mediated by its glycoprotein fraction. Preliminary SDS-PAGE analysis suggested that CA-SIIF protein has a molecular weight of around 70 kDa (data not shown). Further purification and identification of this CA-SIIF glycoprotein is currently underway in our group.

## Discussion

Previous studies have demonstrated that *C*.*albicans *influences immune response (Th1/Th2 balance) patterns during infection [15,16]. Since IL-12 plays a central role in linking the innate and acquired immune systems [5], suppression of this cytokine could be a key to survival of any potential pathogen. IL-12 produced by monocytes/macrophages has been demonstrated to play a central role in the production of IFN-γ by NK and T cells, acting in concert with IL-18, IL-1β, TNF-α and IL-2. Moreover, IL-12 stimulates both NK and T cells to produce cytokines evoking an IFN-γ response. IFN-γ production results in increased cytotoxic activity and enhanced pathogen killing. Conversely, IL-12 suppression may lead to a predominant Th2 type response to *Candida *infections. Our previous studies indicate that the factor(s) responsible for inhibition of HKCA stimulated IL-12 associated with virulent strains of *C. albicans *at serum culture condition, is released into the media as a soluble secretory IL-12 inhibitory factor (CA-SIIF) [8]. In this study, to exclude the possible effect of serum and HKCA on further characterization and purification of this protein, we collected CA-SIIF at serum-free condition and induced IL-12 by IFN-γ priming and LPS stimulation, and compared different doses with media control and another supernatant obtained from the non-pathogenic control, *Saccharomyces cerevisiae*. Our results suggested CA-SIIF is serum-independent but CA-specific, and 200 μg CA-SIIF from serum-free conditions is enough to show a significant inhibitory effect on IL-12 production by 1 × 10^6 ^human monocytes. This finding was used as background for performing further investigations on CA-SIIF with human monocytes.

Macrophages, in many cases, are localized monocytes entering damaged tissue through blood vessel epithelium with a series of changes in morphology and biological functions. We speculate that since human monocytes are suppressed in their ability to produce IL-12 following exposure to CA-SIIF, that this same phenotype can be predicted for murine macrophages as well. Therefore, we established a mouse model to investigate the impact of CA-SIIF on IL-12 production by murine macrophages *in vitro *and *in vivo*. Treatment with LPS and IFN-γ resulted in stimulation of IL-12 production, while *in vitro *addition or *in vivo *injection of CA-SIIF repressed IL-12 production. In this regard, IL-12 production in TG-elicited murine peripheral macrophages was decreased by 32% and 45% *in vitro *and *in vivo *respectively, following CA-SIIF treatment. This demonstrates the same trend as human Monocytes. Human monocytes stimulated by LPS and IFN-γ were inhibited approximately 67% by CA-SIIF, as shown above in Figure 2. The difference in the levels of IL-12 inhibition between murine macrophages and human monocytes might be related to the state of cell differentiation (monocytes versus macrophages). Significantly, these results showed the ability of murine-derived cells to respond to CA-SIIF in a fashion similar to that observed for human immune cells, demonstrating the clinical relevance of our results.

It also has been reported that, when treated with *C. albicans*, peripheral blood mononuclear cells produce MCP-1, RANTES and IL-8 [17,18], and elevations of MCP-1 level in human alveolar macrophages are also induced [19]. Provided IL-12 is inhibited by CA-SIIF, it had been our interest to explore whether CA-SIIF has any inhibitory effect on other pro-inflammatory cytokines and chemokines. Our data shows that addition of CA-SIIF repressed various pro-inflammatory cytokines compared to IFN-γ/LPS-stimulated monocytes alone. Pro-inflammatory cytokines or chemokines such as IL-8, IL-12, MCP-2, MIP-1Δ, RANTES and two generally implicated in allergic responses, IL-13 and Eotaxin-2, were down-regulated by monocytes exposure to CA-SIIF. Also, three cancer-associated proteins, GRO, LIF, TIMP-2 were also down-regulated, along with Leptin, an energy intake and expenditure regulator. It is also worth mentioning that levels of the anti-inflammatory cytokine IL-10 were also reduced significantly, which indicates a complex interaction of cytokines and chemokines in response to fungal infections. Interestingly, a newly reported role for IL-17A in host defense against *C. albicans *infection has also been published [20]. These authors demonstrate that IL-17A plays a critical role in host defense. It is possible, although we did not examine it directly in these studies, that CA-SIIF may also suppress other anti-*Candida *cytokines such as IL-17.

In our study, levels of MCP-4, MIP-3α and especially MIF, a chemokine that inhibits macrophage migration, were up-regulated. Overall, large scale suppression of pro-inflammatory cytokines or chemokines and up-regulation of specific anti-inflammatory factor like MIF suggests *C. albicans*' ability to use CA-SIIF to suppress inflammatory effects of immune cells. Such ability may contribute to possible refractory *Candida *infections in patients. However, whether some of the cytokines or chemokines' differential expression were the result of a larger scale cytokine/chemokine cross-talk remains unknown.

Dendritic cells, which are differentiated from precursor monocytes, express Toll-like receptors and other surface receptors interacting with pathogens, which play an active role in host protection against *Candida *infections, especially in the aspect of antigen presentation [21]. Since ERK and p38 MAPK are involved in CA-SIIF's inhibitory effect [8] and reciprocally regulate the differentiation of monocyte-derived dendritic cells [13], we suspected that the derivation from monocytes to dendritic cells might also be inhibited by CA-SIIF. By measuring cell surface markers specific to monocytes or derived dendritic cells through two color immunofluorescence staining flow cytometry, we found CA-SIIF significantly decreased CD1a expression on monocyte-derived dendritic cells induced by GM-CSF and IL-4. This provides us an increased understanding of other aspects of CA-SIIF inhibitory effect on monocytes, in terms of preventing them from becoming more specific and mature dendritic cells, which are responsible for local antigen presentation and establishment of (Th1) protective immune responses against *Candida *infection. Nevertheless, a recent report discovered that, even though subpopulations of moncoyte-derived dendritic cells (MoDCs) are phenotypically related to CD34 positive stem cell-derived dendritic cells (CD34DCs), MoDCs express a specific integrin VLA-6 but CD34DCs does not. Additionally, the adhesion and binding to components of cutaneous extracellular matrix between the two also differ, which suggests more investigations need to be performed before we draw a simple conclusion of CA-SIIF's effect on other dendritic cells subpopulations [22].

Many biologically functional proteins are glycosylated. Recent study of *C. albicans *secreted proteinaceous materials by proteomic analysis suggests a large portion of glycosylated proteins, of which many are also similar to the components present in cell wall/surface fractions and were generally not considered within the classical *Candida *secretome [23]. In our previous study, we hypothesized and performed preliminary biochemical analysis to show that IL-12 inhibitory activity of CA-SIIF might be due to the presence of a carbohydrate(s) [8]. Thus, in this study, to determine whether CA-SIIF is glycoprotein in nature, we performed one-step purification of CA-SIIF by passing the crude CA-SIIF preparation through commercially-made ConA lectin column. Next, we evaluated the inhibitory effects of fractionated CA-SIIF on monocyte IL-12 production. Our results showed that the ConA-bound glycoprotein fraction had a higher inhibitory efficiency (fold decrease/mg sample), compared to the non-glycoprotein fraction and the crude CA-SIIF preparation. These results clearly demonstrated that the inhibitory activity of CA-SIIF is mediated by glycoprotein(s) rich in high mannose-type and hybrid-type oligosaccharides [14]. This purified CA-SIIF fraction allows us to perform more detailed biochemical and molecular analyses regarding the mechanism of action of this factor in the future.

Several studies have reported β-glucans isolated from *Candida *cell walls to exhibit immunomodulatory activities [24-26]. However, isolation of glucans from *Candida *cell walls involves stringent extraction steps, including treatment with NaClO and dimethylsulfoxide [26], and hot acid and alkali treatments [25,27]. While presence of serum also induces secretion of glucans by *C. albicans *[27], our studies revealed that the IL-12 inhibitory activity of CA-SIIF was retained even in the absence of serum. Furthermore, we found that IL-12 inhibitory activity of CA-SIIF is abrogated by proteinase treatment, indicating this activity is retained in the protein fraction of CA-SIIF and is not due to glucan.

It has been previously shown that, after phagocytosis of *C. albicans *yeast forms, monocytes failed to differentiate to dendritic cells and their IL-12 production was also inhibited. In contrast, while phagocytosis of germ tube forms of *C. albicans *leads to inhibition of IL-12, the maturation from monocytes to dendritic cells remains unaffected [28]. Another study showed that hyphal-form of *C. albicans *can suppress IL-12 production even in the absence of phagocytosis [29]. Results described in the current study, and our previous study showed that IL-12 inhibition can also be achieved by soluble factors secreted by *C. albicans *[8]. The growth medium (RPMI-1640) used in these studies is known to induce hyphal formation and we did see more hyphae when collecting CA-SIIF, suggesting CA-SIIF is possibly secreted more by hyphal-form of *C. albicans*. Since hyphal forms of *C. albicans *are generally associated with increased virulence, it is possible that CA-SIIF could have a larger role in disease, contributing to the pathogenicity of this organism. However, further studies need to be done to confirm this hypothesis. Release of *C. albicans *proteins or molecules that regulate cytokine production is in agreement with other studies. For example, the *C. albicans *Water-Soluble Mannoprotein-β-glucan Complex (CAWS), which resembles the free β-1,3-D-glucan in patient blood, is known to modulate the growth and cytokine production of murine macrophage cell line [30]. A recent study has also found that farnesol pretreatment reduced both IFN-γ and IL-12, but not TNF-α and exhibited IL-5 increase [15].

Taken together, our studies suggest another novel way that *C. albicans *may suppress the immune response, namely by secreting CA-SIIF, which can modulate the Th1 protective immune responses, immune cell differentiation, and inflammatory responses.

## Conclusion

Our results show that the IL-12 inhibitory activity of *C. albicans *is mediated by CA-SIIF, which is a glycoprotein, and serum-independent. Moreover, CA-SIIF inhibits IL-12 production from monocytes *in vitro *and *in vivo*, and can regulate the differentiation of monocytes to dendritic cells. These results suggest that CA-SIIF may play important roles in interactions between *C. albicans *and the host immune system.

## Authors' contributions

MW carried out purification and characterization of CA-SIIF and evaluation of its effect on monocyte IL-12 production and differentiation, and wrote early drafts of the manuscript. PKM designed the experiments, evaluated results and helped to write the manuscript. MW and JC carried out the cytokine array studies. AAL participated in gel electrophoresis-based characterization and affinity purification of CA-SIIF. TSM designed the studies describing *in vitro *and *in vivo *activation of monocytes and their exposure to CA-SIIF. MAG designed the study, and participated in its design and coordination and edited the manuscript. All authors read and approved the final manuscript.
